# Effects of glucocorticoid pulse therapy on thyroid function and thyroid antibodies in children with graves’ disease

**DOI:** 10.1186/s13052-021-00999-5

**Published:** 2021-03-02

**Authors:** Yanyan Hu, Yulin Man, Xuemei Sun, Yongzhen Xue

**Affiliations:** 1 Department of Pediatrics, Linyi People’s Hospital, NO.27, Eastern Jiefang Road, Linyi, 276000 Shandong Province China; 2 Department of Nephrology, Linyi People’s Hospital, NO.27, Eastern Jiefang Road, Linyi, 276000 Shandong Province China

**Keywords:** Glucocorticoid pulse therapy, Graves’ disease, Thyroid function, Thyroid antibodies, Children

## Abstract

**Background:**

Glucocorticoid treatment is used in children with Graves’ disease (GD) only in cases of exophthalmos. The purpose of this study was to observe the effects of glucocorticoid pulse therapy on thyroid function and thyroid antibodies in children with GD.

**Methods:**

Twenty children who were treated by intravenous methylprednisolone pulse therapy (MPT) followed by oral prednisolone administration and antithyroid drugs were included in the pulse group. Twenty children who were treated with antithyroid drugs alone were included in the control group. Serum concentrations of free triiodothyronine (FT3), free thyroxine (FT4), thyrotropin (TSH), thyroid peroxidase antibodies (TPOAb), thyroglobulin antibodies (TGAb), and thyrotropin receptor antibodies (TRAb) were recorded at baseline and 10 days, 30 days, and 60 days after treatment.

**Results:**

Significant differences in FT3, FT4, TSH, TPOAb, TGAb, and TRAb levels were found in the pulse group and the control group from baseline to follow-up time points (all *p* < 0.05). On the 30th day, the TRAb level in the pulse group was significantly lower than that in the control group (*p* = 0.023). However, the level of TRAb rose on the 60th day. For values of TRAb at baseline, 10 days, and 60 days after treatment, there were no significant differences respectively between the pulse group and the control group (all *p* > 0.05). No significant differences were observed in FT3, FT4, TSH, TPOAb, and TGAb levels between the pulse group and the control group (all *p* > 0.05).

**Conclusions:**

The results suggested that the effect of intravenous MPT followed by oral prednisolone on TRAb level was temporary in children with GD. Glucocorticoid pulse therapy was not beneficial for the sustained recovery of thyroid function.

## Background

Hyperthyroidism is a relatively rare disease in children compared to adults [1]. The incidence rate of hyperthyroidism in children is increasing over the last decades [2]. The most common cause of hyperthyroidism in children is Graves’ disease (GD). GD is more common in children with Down or Turner syndrome (TS) than in the general population [3–5]. A wide variety of clinical manifestations in GD have been described. Some individuals even progress from Hashimoto’s thyroiditis (HT) to GD [6]. GD has serious adverse effects on children’s growth and development [7].

The purpose of treatment for GD is not only to reduce the excess production of thyroid hormone and maintain normal thyroid function but also to prevent recurrence of hyperthyroidism. Seeking the most appropriate therapy for children is very important and urgent. Antithyroid drugs (ATD) therapy is usually the initial treatment for children with GD at present. However, uncontrolled symptoms, long-term remission, and high recurrence rate are often presented in children with GD who only use antithyroid drugs. How to correct thyrotoxicosis promptly and maintain euthyroidism stably for children with GD should be solved. Glucocorticoid treatment is used in children with GD only in cases of exophthalmos [8, 9] A novel use has been explored in this study. The purpose of this study was to observe the effects of intravenous methylprednisolone pulse therapy (MPT) followed by oral prednisolone administration on thyroid function and thyroid antibodies in children with GD.

## Materials and methods

### Patients

The retrospective study included children who were newly diagnosed with GD between 2014 to 2018 at our Pediatric Department. The including criteria were as follows: the diagnosis of GD was based on clinical and biochemical findings, such as palpitations, fatigue, irritability, increased appetite, weight loss, diarrhoea, increased perspiration, elevated serum free thyroxine (FT4) and free triiodothyronine (FT3) levels, suppressed serum thyrotropin (TSH) level, and positive thyrotropin receptor antibodies (TRAb); the presence of bilateral exophthalmos according to the reference [10]. Children with peptic ulcers, severe hypertension, cardiovascular diseases, liver dysfunction, tumor, diabetic mellitus, infectious diseases, and other autoimmune diseases were excluded. This study also excluded children who had received previous ATD treatment, and those who were lost to follow-up. A total of 40 children fulfilled the criteria. Of these, 20 children were treated by intravenous MPT followed by oral prednisolone administration and ATD treatment, aged 7.7 ± 3.2 (range 2.0–14.0) years, 6 boys and 14 girls, 20 children were treated with ATD alone, aged 8.9 ± 4.0 (range 2.0–14.0) years, 4 boys and 16 girls. The control group and the pulse group had similar age and sex distribution.

### Study protocol

Clinical and treatment data were obtained from children’s medical records. Age, sex, clinical signs, goiter size, serum concentrations of FT3, FT4, TSH, thyroid peroxidase antibodies (TPOAb), thyroglobulin antibodies (TGAb), and TRAb, electrocardiogram (ECG), and thyroid and cardiac ultrasound were recorded at diagnosis and during follow-up. Follow-up visits occurred at 10 days, 30 days, and 60 days after treatment.

All children received ATD treatment (methimazole 1.0 mg/kg/day) after diagnosis. The dose of methimazole was adjusted according to individual’s weight and thyroid hormone levels. Metoprolol (0.5–1.0 mg/kg/day) was administered orally if heart rate exceeds 100 bpm [11]. In the pulse group, high-dose methylprednisolone was infused intravenously at a dose of 5 mg/kg/day for 3 successive days, and the dosage was reduced by half every 3 days. Blood pressure, blood glucose, and ECG were monitored daily. After 3 cycles of this course, oral prednisolone was administered. The dosage of prednisolone was gradually tapered off over a period of 3 weeks. Oral calcium and vitamin-D were also supplemented for children during glucocorticoid treatment.

The study was approved by the Ethics Committee of Linyi People’s Hospital (approval number: YX10070). Written informed consent was obtained from children’s parents.

## Methods

Fasting serum samples were collected in the morning from all subjects. Serum FT3, FT4, TSH, TPOAb, TGAb, and TRAb concentrations were measured by an automated chemiluminescence immunoassay system (Advia Center, Siemens, Munich, Germany). Normal ranges were 2.27–4.22 pg/mL for FT3, 0.88–1.75 ng/dL for FT4, 0.35–5.5 μIU/mL for TSH, 0–60 IU/mL for TPOAb, 0–60 IU/mL for TGAb, and 0–1.22 IU/L for TRAb. TPOAb, TGAb, and TRAb values above the normal ranges were defined as positive. The intra- and inter-assay coefficients of variation were all < 5.0 and < 4.0%, respectively. The sensitivity of FT3, FT4, TSH, TPOAb, TGAb, and TRAb were 0.19 pg/mL, 0.1 ng/dL, 0.001 μIU/mL, 0.1 IU/mL, 10 IU/mL, and 0.01 IU/L, respectively.

Grading of goiter was performed by inspection and palpation, and was classified according to the World Health Organization (WHO) criteria: grade 0, no goiter; grade 1, goiter palpable but not visible; grade 2, goiter visible with neck in normal position [12]. Degree of proptosis was measured with a Hertel exophthalmometer. Physical examination of all subjects was done by one experienced investigator.

Thyroid ultrasound was performed with a 5–12 MHz linear-array transducer on a LOGIQ 9 scanner (GE Medical Systems, Milwaukee, WI, USA). Echocardiography was performed with a 2–5 MHz transducer using an IU-22 ultrasound scanner (Philips Medical System, Bothell, WA, USA). All ultrasound examinations were carried out by the same trained sonographer. ECG was recorded with an electrocardiograph (1550p; Nihon Kohden, Tokyo, Japan) at paper speed of 25 mm/s and 10 mv/mm.

### Statistical analysis

Normal distribution of variables was checked with the Shapiro-Wilk test before further analyses. Values of FT3, FT4, and TRAb showed Normal distributions. Values of TSH, TPOAb, and TGAb showed skewed distributions. Therefore, normally distributed variables were expressed as mean ± standard deviation, and skewed distributed data were expressed as the median and interquartile range. Chi-square test was used to compare clinical characteristics of the pulse group and the control group. Repeated measures general linear models were used to determine whether there were changes in values of FT3, FT4, and TRAb at baseline, 10 days, 30 days, and 60 days. Friedman’s nonparametric test was used to analyze changes in TSH, TPOAb, and TGAb levels at different time points. The Mann-Whitney U test was used to compare nonparametric data at each moment between groups. A level of *p* < 0.05 was considered statistically significant. The analyses were performed using statistical software SPSS version 19.0 (SPSS Inc. Chicago, USA).

## Results

### Baseline clinical characteristics

In the pulse group, the duration of clinical symptoms was about 0.50(0.18, 1.00) years. In the control group, the duration of clinical symptoms was about 0.50(0.27, 1.00) years. There was no significant difference in the duration of hyperthyroidism between pulse group and the control group (*p* > 0.05). At the beginning of the study, all children presented with typical symptoms of GD. There were no significant differences in the symptoms of hyperthyroidism, the grading of goiter, heart rate, and blood pressure between the pulse group and the control group (all *p* > 0.05). Baseline clinical characteristics are shown in Table 1.

**Table 1:** Baseline Characteristics of Children with Graves’ Disease

Characteristic	The pulse group (***N*** = 20)	The control group (***N*** = 20)	***P*** value
Gender [male(%)/female(%)]	6 (30.0)/14 (70.0)	4 (20.0)/16 (80.0)	0.465
Age (year)	7.7 ± 3.2	8.9 ± 4.0	0.303
Duration (year)	0.50 (0.18,1.00)	0.50 (0.27,1.00)	0.901
Palpitations(%)	8 (40.0)	10 (50.0)	0.525
Fatigue (%)	9 (45.0)	10 (50.0)	0.752
Irritability (%)	16 (80.0)	15 (75.0)	0.705
Increased appetite (%)	11 (55.0)	9 (45.0)	0.527
Weight loss (%)	3 (15.0)	4 (20.0)	0.677
Diarrhoea (%)	7 (35.0)	9 (45.0)	0.519
Increased perspiration (%)	11 (55.0)	12 (60.0)	0.749
Exophthalmos (mm)	17.85 ± 4.09	18.25 ± 4.36	0.767
Goiter grade 1 (%)	5 (25.0)	4 (20.0)	0.705
Goiter grade 2 (%)	15 (75.0)	16 (80.0)	0.705
Heart rate (bpm)	114 ± 18	115 ± 17	0.873
Systolic blood pressure (mmHg)	121 ± 12	124 ± 11	0.449
Diastolic blood pressure (mmHg)	71 ± 4	74 ± 5	0.063
TPOAb(+) (%)	17 (85.0)	17 (85.0)	1.000
TGAb(+) (%)	15 (75.0)	14 (70.0)	0.723
TRAb(+) (%)	20 (100.0)	19 (95.0)	0.311

Data are presented as means±SD, median (interquartile range), or number (percentage)

*Abbreviations*: *TPOAb* thyroid peroxidase antibodies, *TGAb* thyroglobulin antibodies, *TRAb* thyroid stimulating hormone receptor antibodies

### Laboratory and imaging parameters before treatment

There were no significant differences in the values of FT3, FT4, TSH, TPOAb, TGAb, and TRAb between the pulse group and the control group (all *p* < 0.05). The parameters of TPOAb, TGAb, and TRAb before treatment are shown in Table 1. Ultrasound examinations of thyroid showed increased thyroid volume and parenchymal blood flow in all children. In the pulse group, there was 1 child who had thyroid nodules, and 1 child who had thyroid follicular cysts. Cardiac ultrasound examinations revealed that 4 children had enlarged left atrium and left ventricle and mitral regurgitation, the others showed normal results. In the control group, cardiac ultrasound examinations revealed that 1 child had enlarged left atrium and ventricle and mitral regurgitation, the others showed normal results. The ECG in the pulse group showed that 11 children had sinus tachycardia, 1 child had excessive left ventricular voltage, 3 children had T-wave changes, 1 child had sinus arrhythmia, and 4 children were normal. The ECG in the control group showed that 11 children had sinus tachycardia, 2 children had sinus arrhythmia, and 7 children were normal.

### Clinical, laboratory, and imaging parameters after treatment

In analysis using repeated measures of FT3, FT4, and TRAb levels over time, there was significant difference from baseline to follow-up time points (all *p* < 0.001). FT3, FT4, and TRAb levels at different time points were compared respectively.

In the pulse group and the control group, there were significant differences in FT3 levels between baseline and 10, 30, and 60 days after treatment (all *p* < 0.001). However, there were no significant differences among 10, 30, and 60 days after treatment (all *p* > 0.05). The group by time interaction for FT3 levels was not significant (*p* = 0.132). There was no significant difference between the pulse group and the control group (*p* = 0.414).

In the pulse group, there were significant differences in FT4 levels between baseline and 10, 30, and 60 days after treatment (all *p* < 0.01), there were significant differences between 10 days and 30, and 60 days after treatment (all *p* < 0.001), and there was no significant difference between 30 and 60 days after treatment (*p* = 0.705). In the control group, the results showed that there were significant differences between baseline and 10, 30, and 60 days after treatment (all *p* < 0.01), and there were no significant differences among 10, 30, and 60 days after treatment (all *p* > 0.05). The group by time interaction for FT4 levels was not significant (*p* = 0.186). There was no significant difference between the pulse group and the control group (*p* = 0.549).

In the pulse group, there were significant differences in TRAb levels between baseline and 10, 30, and 60 days after treatment (all *p* < 0.01), there were significant differences between 10 days and 30, and 60 days after treatment (all *p* < 0.05), and there was no significant difference between 30 and 60 days after treatment (*p* = 0.846). In the control group, the results showed that there were significant differences between baseline and 30, and 60 days after treatment (all *p* < 0.05), and there were no significant differences among 10, 30, and 60 days after treatment (all *p* > 0.05). The group by time interaction for TRAb levels was significant (*p* = 0.001). For values of TRAb at baseline, 10 days, and 60 days after treatment, there were no significant differences between the pulse group and the control group (all *p* > 0.05), but for values of TRAb at 30 days, there was significant difference between the pulse group and the control group (*p* = 0.023).

**Fig. 1:**
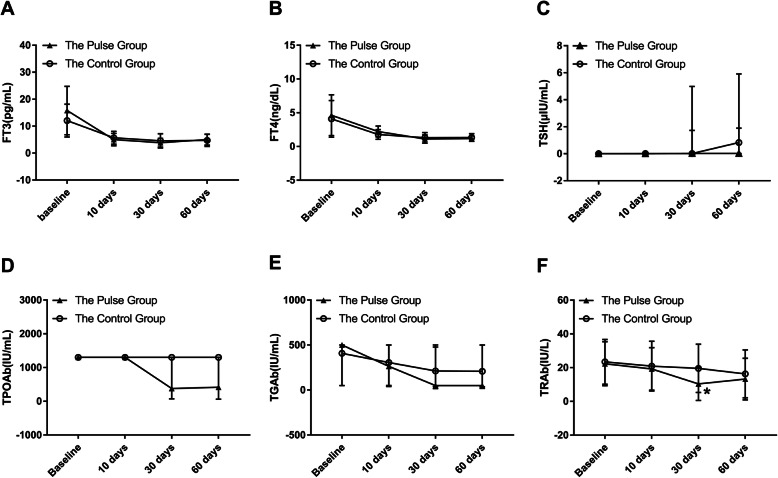
The Changes of Thyroid Function and Thyroid Antibodies at Baseline and 10 days, 30 days, and 60 days after Treatment in Children with Graves’ Disease (The pulse group, black triangle; the control group, white circle). Data are presented as means±SD in values of FT3, FT4, and TRAb, and median (interquartile range) in values of TSH, TPOAb, and TGAb. * *P* < 0.05 for the pulse group vs. the control group. Abbreviations: FT3, free triiodothyronine; FT4, free thyroxine; TSH, thyroid stimulating hormone; TPOAb, thyroid peroxidase antibodies; TGAb, thyroglobulin antibodies; TRAb, thyroid stimulating hormone receptor antibodies

There were no significant differences between the pulse group and the control group in the values of TSH, TPOAb, and TGAb (all *p* > 0.05). TSH, TPOAb, and TGAb levels at different time points were compared respectively. In the pulse group, the results showed that there were significant differences in TSH, TPOAb, and TGAb levels from baseline to follow-up time points (*p* = 0.001, *p* = 0.001, and *p* = 0.002, respectively). In the control group, there were significant differences in TSH, TPOAb, and TGAb levels from baseline to follow-up time points (*p* < 0.001, *p* = 0.036, and *p* = 0.001, respectively). The changes of thyroid function and thyroid antibodies in children with GD are shown in Fig. 1.

In the pulse group, 7 children had elevated fasting blood glucose temporarily, 5 children had mild hypertensive on the basis of hyperthyroidism. After treatment, the above symptoms returned to normal. The clinical characteristics after treatment are shown in Table 2. The children in this study still showed exophthalmos and thyroid goiter after 60 days of treatment. The abnormal of typical symptoms of hyperthyroidism, cardiac ultrasound, and ECG were all relieved in all children.

**Table 2:** Characteristics of Children with Graves’ Disease after Treatment

Characteristic	The pulse group (***N*** = 20)	The control group (***N*** = 20)	***P*** value
Palpitations (%)	3 (15.0)	1 (5.0)	0.292
Fatigue (%)	3 (15.0)	2 (10.0)	0.633
Irritability (%)	8 (40.0)	10 (50.0)	0.525
Increased appetite (%)	17 (85.0)	1 (5.0)	< 0.001*
Weight loss (%)	0 (0)	0 (0)	1.000
Diarrhoea (%)	1 (5.0)	2 (10.0)	0.548
Increased perspiration (%)	8 (40.0)	5 (25.0)	0.311
Exophthalmos (mm)	17.73 ± 3.92	18.18 ± 4.25	0.730
Heart rate (bpm)	90 ± 8	87 ± 9	0.341
Systolic blood pressure (mmHg)	100 ± 7	98 ± 5	0.426
Diastolic blood pressure (mmHg)	64 ± 3	65 ± 4	0.661
TPOAb(+) (%)	15 (75.0)	16 (80.0)	0.705
TGAb(+) (%)	8 (40.0)	15 (75.0)	0.025*
TRAb(+) (%)	20 (100.0)	18 (90.0)	0.147

Data are presented as means±SD, median (interquartile range), or number (percentage)

No change occurred in exophthalmos and thyroid goiter after 60 days of treatment

*Abbreviations*: *TPOAb* thyroid peroxidase antibodies, *TGAb* thyroglobulin antibodies, *TRAb* thyroid stimulating hormone receptor antibodies

*P < 0.05

## Discussion

In this study, the effects of intravenous MPT followed by oral prednisolone administration on thyroid function and thyroid antibodies were observed in children with GD. We found that no significant differences in FT3, FT4, TSH, TPOAb, and TGAb levels between the pulse group and the control group. On the 30th day, the TRAb level in the pulse group was significantly lower than that in the control group. However, the level of TRAb rose on the 60th day, and there was no difference between the pulse group and the control group. The results indicated that the intravenous MPT followed by oral prednisolone administration had a transient mitigation effect on TRAb level in children with GD.

GD is an autoimmune thyroid disease. Genetic predisposition and environmental factors contribute to the development of autoimmune thyroid disease. Polymorphism of genes occurs in genetically predisposed individuals [13]. Deficiency in suppressor T cells and loss of self-tolerance are mainly involved in the pathogenesis of GD [14]. Both cell-mediated and humoral responses lead to high levels of TRAb [15]. TRAb binds to TSH receptor (TSHR) to activate thyroid gland, leading to hyperthyroidism and goiter. Excess circulating thyroid hormone causes a wide range of clinical symptoms. However, many patients have antibodies against TSHR and thyroid peroxidase, and about 50% of them have antibodies against thyroglobulin [16]. In this study, most of children showed positive TRAb, TGAb and TPOAb.

Glucocorticoids have immunoregulatory effects. It could decrease aggregation of macrophage, interfere with function of T cells and B cells, reduce the activity of immune cells, inhibit the release of inflammatory mediators (i.e. cytokines and prostaglandins), lower the concentration of thyroid-stimulation immunoglobulins, and prevent antibodies from binding to receptors [17, 18]. In addition, glucocorticoids have non-specific anti-inflammatory effects. It could reduce interstitial capillary dilatation, exudation and edema, inhibit the proliferation of vascular endothelial cells, alleviate interstitial congestion, and decrease thyroid gland volume. Therefore, glucocorticoids could control hyperthyroidism to some extent.

The mechanism for glucocorticoids used in GD is their immunosuppressive and anti-inflammatory effects. Glucocorticoids could be given orally, intravenously and locally [19]. Retrobulbar injection of glucocorticoids may be considered under circumstances of absolute contraindications to systemic glucocorticoids [20]. Therefore, intravenous and oral glucocorticoids were used in this study.

The effect of glucocorticoids therapy on Graves’ orbitopathy has been studied in most studies. A meta-analysis of 14 clinical trials showed that glucocorticoids were more effective than other treatments for Graves’ orbitopathy, especially the intravenous glucocorticoid pulse therapy [21]. A prospective, randomized, and placebo-controlled study also reported that MPT effectively improved diplopia, eye movement and proptosis for active and moderately severe Graves’ orbitopathy. It was well tolerated and safe. However, the proptosis of one eye worsened in 1 patient. The effect of treating proptosis with MPT was not so obvious for overall outcome of both eyes [22]. A small number of paediatricians recommended the use of glucocorticoids in children with moderate-to-severe and active Graves’ orbitopathy. Glucocorticoids are the choice of treatment [8]. Based on this recommendation, GD children associated with orbitopathy were treated with MPT treatment in this study. However, the difference of proptosis in children with GD was not noted after MPT treatment. This may be due to our short-term follow-up.

Few studies have been conducted on the effects of glucocorticoids therapy on thyroid function and thyroid antibodies. A study found that the level of TSH binding inhibitor immunoglobulin (TBII) in some adult patients with GD decreased significantly after 3 to 6 months of pulse therapy, but increased again at 12 to 24 months. The effect of pulse therapy on autoimmune antibody was transient. The pulse therapy did not improve remission rate of GD [23]. However, studies of the effects of glucocorticoids therapy on thyroid function and thyroid antibodies in GD children are few. Therefore, glucocorticoids therapy on thyroid function and thyroid antibodies in GD children is our main observation. There is no clinical evidence on the optimal dosage and regimen of intravenous and oral glucocorticoids for children. Prolonged glucocorticoid administration will lead to Cushing’s syndrome, hyperglycemia, hypertension, gastrointestinal ulcer, infection, osteoporosis, growth retardation, etc. [24]. Therefore, based on the above considerations, intravenous MPT followed by oral prednisolone administration was used in this study. After 9 days of MPT, oral prednisolone was administered. The dosage of prednisolone was gradually tapered off over a period of 3 weeks. Increased appetite, hyperglycemia, and hypertension were found in some of children in the pulse group. After ceasing glucocorticoid therapy, these symptoms returned to normal. It could reduce the side effects of prolonged glucocorticoid administration to some extent.

In this study, the TRAb level on the 30th day in the pulse group was significantly lower than that in the control group. It was due to the immunosuppressive effect of glucocorticoids. The level of TRAb rose on the 60th day, and there was no difference between the pulse group and the control group. It was due to the discontinuation of glucocorticoids after 30 days. In addition, the rate of positive TGAb in the pulse group was lower than that in the control group also due to the immunosuppressive effect of glucocorticoids. There was no difference in the level of TGAb between the pulse group and the control group. It may be due to our small sample size.

There are two main limitations in this study. On one hand, different dosage and regimen of glucocorticoid administration may result in different therapeutic effects. There are no literatures which clearly specify the dosage and regimen of glucocorticoid administration for children. Considering the side effects of glucocorticoids on children, we adopted small dosage of intravenous MPT followed by oral glucocorticoids. On the other hand, this is a retrospective cohort study based on clinical practice with consents of parents and ethics committee. The clinical practice provided initial exploration for follow-up prospective study. The sample size was small and the observation time was short in this study. In fact, GD is relatively rare in children. The half-life of glucocorticoid is relatively short.

## Conclusion

In conclusion, the effect of intravenous MPT followed by oral prednisolone on TRAb level was temporary in children with GD. Glucocorticoid pulse therapy was not beneficial for the sustained recovery of thyroid function. In the future, the dosage and regimen of glucocorticoid administration, as well as new treatments which could reduce the recurrence rate and restore thyroid function promptly need to be further explored for children with GD.

## Data Availability

All data generated or analysed during this study are included in this published article.

## Abbreviations

GD: Graves’ disease
MPT: Methylprednisolone pulse therapy
FT3: Free triiodothyronine
FT4: Free thyroxine
TSH: Thyroid stimulating hormone
TPOAb: Thyroid peroxidase antibodies
TGAb: Thyroglobulin antibodies
TRAb: Thyroid stimulating hormone receptor antibodies

## References

[1] De Luca F, Alessi L, Bruno E, Cama V, Costanzo D, Genovese C (2016). Graves’ disease in childhood: new epidemiological, pathophysiological and therapeutic insights. Minerva Pediatr.

[2] Kjær RH, Andersen MS, Hansen D (2015). Increasing incidence of juvenile thyrotoxicosis in Denmark:a nationwide study, 1998-2012. Horm Res Paediatr.

[3] De Luca F, Corrias A, Salerno M, Wasniewska M, Gastaldi R, Cassio A (2010). Peculiarities of Graves' disease in children and adolescents with Down's syndrome. Eur J Endocrinol.

[4] Valenzise M, Aversa T, Corrias A, Mazzanti L, Cappa M, Ubertini G (2014). Epidemiology, presentation and long-term evolution of Graves' disease in children, adolescents and young adults with turner syndrome. Horm Res Paediatr..

[5] Aversa T, Lombardo F, Corrias A, Salerno M, De Luca F, Wasniewska M (2014). In young patients with turner or Down syndrome, Graves' disease presentation is often preceded by Hashimoto’s thyroiditis. Thyroid..

[6] Wasniewska M, Corrias A, Arrigo T, Lombardo F, Salerno M, Mussa A (2010). Frequency of Hashimoto's thyroiditis antecedents in the history of children and adolescents with graves' disease. Horm Res Paediatr..

[7] Srinivasan S, Misra M (2015). Hyperthyroidism in children. Pediatr Rev.

[8] Krassas GE, Segni M, Wiersinga WM (2005). Childhood Graves' ophthalmopathy: results of a European questionnaire study. Eur J Endocrinol.

[9] Penta L, Muzi G, Cofini M, Leonardi A, Lanciotti L, Esposito S (2019). Corticosteroids in moderate-to-severe Graves' Ophthalmopathy: Oral or intravenous therapy?. Int J Environ Res Public Health.

[10] Mingzhi Z, Rongzhao H, Zhifu FU (2000). The measurement of normal values of exophthalmos, interpupillary distance and interorbital distance of children and adolescence in Xiamen and the rule of their development. Chin J Ophthalmol.

[11] Bahn Chair RS, Burch HB, Cooper DS, Garber JR, Greenlee MC, Klein I (2011). Hyperthyroidism and other causes of thyrotoxicosis: management guidelines of the American Thyroid Association and American Association of Clinical Endocrinologists. Thyroid..

[12] WHO U. Assessment of the iodine deficiency disorders and monitoring their elimination. Geneva. 2001:1–107.

[13] Sawicka B, Borysewicz-Sańczyk H, Wawrusiewicz-Kurylonek N, Aversa T, Corica D, Gościk J (2020). Analysis of Polymorphisms rs7093069-IL-2RA, rs7138803-FAIM2, and rs1748033-PADI4 in the Group of Adolescents With Autoimmune Thyroid Diseases. Front Endocrinol (Lausanne).

[14] Ramos-Leví AM, Marazuela M (2016). Pathogenesis of thyroid autoimmune disease: the role of cellular mechanisms. Endocrinol Nutr.

[15] Rydzewska M, Jaromin M, Pasierowska IE, Stożek K, Bossowski A (2018). Role of the T and B lymphocytes in pathogenesis of autoimmune thyroid diseases. Thyroid Res.

[16] Prabhakar BS, Bahn RS, Smith TJ (2003). Current perspective on the pathogenesis of graves’ disease and ophthalmopathy. Endocr Rev.

[17] Zang S, Kahaly GJ (2011). Steroids and the immune response in Graves’ orbitopathy. Agents Med Chem.

[18] Kahaly G, Bang H, Berg W, Dittmar M (2005). Alpha-fodrin as a putative autoantigen in graves’ ophthalmopathy. Clin Exp Immunol.

[19] Tu X, Dong Y, Zhang H, Su Q. Corticosteroids for Graves' Ophthalmopathy: systematic review and meta-analysis. Biomed Res Int. 2018:4845894. 10.1155/2018/4845894 PMC6282115 30596092

[20] Bartalena L (2013). Graves' orbitopathy: imperfect treatments for a rare disease. Eur Thyroid J.

[21] Abalkhail S, Doi SA, Al-Shoumer KA (2003). The use of corticosteroids versus other treatments for graves’ ophthalmopathy: a quantitative evaluation. Med Sci Monit.

[22] Van Geest RJ, Sasim IV, Koppeschaar HP, Kalmann R, Stravers SN, Bijlsma WR (2008). Methylprednisolone pulse therapy for patients with moderately severe Graves' orbitopathy: a prospective, randomized, placebo-controlled study. Eur J Endocrinol.

[23] Kubota S, Ohye H, Nishihara E, Kudo T, Ito M, Fukata S (2005). Effect of high dose methylprednisolone pulse therapy followed by oral prednisolone administration on the production of anti-TSH receptor antibodies and clinical outcome in Graves' disease. Endocr J.

[24] Zang S, Ponto KA, Kahaly GJ (2011). Clinical review: intravenous glucocorticoids for Graves' orbitopathy: efficacy and morbidity. J Clin Endocrinol Metab.

